# Chronic Heart Disease

**Published:** 1855-02

**Authors:** Alexander Harvey

**Affiliations:** Physician to the Aberdeen Royal Infirmary, &c.


Chronic Heart Disease. By Alexander Harvey, Physician to the Aberdeen Royal Infirmary, &c.—In the following notes, an attempt is made to exhibit, within as small a compass as possible and in a form approaching the tabular, the leading facts connected with the pathology, diagnosis, and prognosis, of the more common organic affections of the heart. Imperfect as the notes are, the author is not without hope that they may be of service to some of his colleagues in the Association, who, though familiar with the subject of heart-disease, may not always be able at once or readily to call to mind such particulars respecting it as they must often have occasion for in the course of their practice, and which they may not always have time or opportunity to collect from large treatises.

It may be added, that the notes were originally drawn up for the students attending the author's class, and delivered to them as a recapitulation of the facts just previously detailed at greater length in the lectures; and that the materials of them are chiefly taken from Dr. Watson's Lectures and Dr. Allison's Outlines of Pathology and Practice of Medicine, the worgs used in the class as text-books.

---

### *Diseases of Valves and Orifices.*

I. The valves and orifices of the left side of the heart are at least twenty times more liable to disease than the valves and orifices of the right side.

Disease of the semilunar pulmonary valves is so rare as to be matter of pathological interest merely. Disease of the tricuspid valves is more frequently, although still seldom, met with; and still seldomer without co-existing disease of either the aortic or mitral valves.

II. In early life, and in women, the mitral valve and corresponding auriculo-ventricular orifice are oftenest diseased; in advanced life, and in men, the aortic. In many cases, at all ages and in both sexes, the two sets of valves and openings are simultaneously diseases.

III. The diseased states of the valves and orifices are mostly the result of antecedent endocarditis of rheumatic origin, and consist of concretions forming on the valves; or mere perversion of nutrition, independent of inflammation, and occurring in connexion with atheromatous and other kinds of deposit in the aorta and arteries generally.

IV. The effects of these changes on the functions of the valves and orifices are two-fold:—

1. By contracting and narrowing the orifice, to impede or obstruct the passage of the blood (*valvular obstruction*, *obstructive disease of valves*).

2. By thickening, corrugating, and shortening the valves, to make the orifice more or less permanently patent, and allow regurgitation or reflux of blood (*valvular insufficiency*, *valvular patency*, *regurgitant disease of valves*).

V. Valvular obstruction and valvular insufficiency often exist separately, but are oftener co-existent in the same valve. Each of them may obtain in very different degrees, and the two may be very variously combined.

VI. In a very few cases, the cause of patency is preternatural widening of the orifice without disease, but with no adequate extension by growth, or the valves; and, in a few others, it is owing to disease with shortening of the chordæ tendineæ, the valves themselves being sound.

---

### *Diseases of Walls and Cavities.*

I. Hypertrophy and dilatation may be either partial or general, oftenest partial. Dilatation, unaccompanied by hypertrophy, is rare.

II. Hypertrophy and dilatation are very generally *secondary* morbid states. They may be produced by various causes:—

1. In a very few cases, by unknown causes, or causes unconnected with obvious change of structure elsewhere (*primary*).

2. By adhesion of the pericardium to the heart, consequent on plastic exudation from pericarditis.

3. By preternatural widening, with or without change of structure, of the ascending aorta.

4. By diseased states of the valves. This is the most common cause.

III. The *rationale* of their production may be thus stated:—

1. Disease of the valves, widening of the aorta, adhesion of the pericardium, create a demand for increased action of the heart; and this increased action, in a constitution otherwide healthy, leads to increased nutrition of the organ. The walls of the heart are thereby gradually increased in bulk and in contractile power (*simple hypertrophy*).

2. When, along with this increased action, there is a full quantity of blood in the body, the cavities of the heart are gradually dilated at the same time that their walls are thickened (*hypertro-*

*phy with dilatation, eccentric hypertrophy*). But when the quantity of circulating blood is much diminished, the tonic contraction of the thickened fibres of the heart, which are no longer distended, leads to a diminution in the size of its cavities (*concentric hypertrophy*). This is rarely met with, except either as a congenital affection, or, in connection with the *rigor mortis*, as a temporary *post mortem* condition.

3. Dilatation without hypertrophy, consequent on obstructive disease of the valves, occurs chiefly in a weakly habit of body, when the excitement of the heart by the preternatural amount of its stimulus is feeble; but it may be produced, in a habit otherwise healthy, by mere morbid debility of the muscular fibres of the heart, as after some cases of rheumatism affecting them.

IV. In the great majority of cases it is the left ventricle that is the first and chiefly affected, and hypertrophy and dilatation combined (eccentric hypertrophy) that obtains, and in connexion with pre-existing mitral or aortic disease. With mitral disease, there is commonly rounding of the apex, and often simple hypertrophy merely; with aortic disease, there is elongation of the apex, and commonly eccentric hypertrophy.

Pure dilatation may affect either ventricle, oftenest, perhaps, the right, and that chiefly in females, and in such cases seems to occur in connection rather with disease of lung (*e. g.* habitual asthma) than with diseased valves.

---

### *Diagnosis of Chronic Heart Disease.*

*Diagnosis of Disease of Valves and Orifices.*—Practically, in at least nineteen out of twenty cases, the questions to be determined are whether it be the mitral or aortic valve that is diseased or both; and whether the disease be of the nature of valvular obstruction, or of valvular insufficiency, or both. The diagnosis is mainly founded on these two things:—

1. A bruit or murmur heard, called, from its blowing character, *bellows murmur*, or, from its seat being within the heart, *endocardial* murmur.

2. The state and character of the pulse at the wrist.

I. *Bruit or murmur.* -Its import varies according to the time when it is heard, the place where it is best heard, and the direction in which it is conveyed furthest. The second and third may be taken together, and included under one head.

*The time when it is heard.*—This may be either with (or rather in place of) the first natural sound (systolic bruit); blood then passing from the left ventricle into either the aorta before, or the auricle behind; or with the second natural sound (diastolic bruit); blood then passing into the ventricle, from either the aorta before, or the auricle behind.

*The place and direction in which it is best heard and conveyed furthest.*—This may be either over the base of the heart, and thence upwards along the aorta and carotids, or at and in the direction of the apex. Accordingly—

1. Bruit, systolic and loudest at base, indicates obstructive disease ot the aortic valves or orifice.

2. Bruit, systolic and loudest at apex, indicates mitral insufficiency (patency) with regurgitation.

3. Bruit, diastolic and loudest at base, indicates aortic insufficience with regurgitation.

4. Bruit, diastolic and loudest at apex, indicates mitral obstruction. Rarely if ever heard.

*** Various combinations of these murmurs are sometimes heard, constituting double bruits. Their import is to be gathered from that of the single murmurs. Thus, *e. g.* there may be a systolic burit, equally loud at the base and at the apex, indicating at once aortic obstruction and mitral insufficiency; or there may be a systolic and a diastolic bruit, both loudest at the base, and indicating aortic obstruction and aortic regurgitation.

II. *The Pulse.*

1. Pulse at wrist irregular, intermittent, unequal and soft, small, or weak, indicates mitral disease, obstructive or regurgitant, particularly the latter, on which this condition of the pulse is specially dependent.

2. Pulse regular, full, or hard, indicates aortic disease; if jerking and resilient, aortic insufficiency (with hypertrophy).

The foregoing (I. and II.) may be thus briefly represented on a card carried in the pocket.

Bruit: If *systolic* and loudest at
*Base*=Aortic obstruction.
*Apex*=Mitral insufficiency.

Bruit: If *diastolic* and loudest at
*Base*=Aortic insufficiency.
*Apex*=Mitral obstruction.

And on the reverse side of the card:—

| | |
|---|---|
| Pulse: If *regular*,<br>Full, or strong,<br>Jerking, resilient, | Aortic disease. |
| Pulse: If *irregular*,<br>Intermittent, unequal,<br>Soft, small, weak. | Mitral disease. |

*Diagnosis of Disease of Walls and Cavities.*

I. Of Hypertrophy.

1. The action of the heart preternaturally, sometimes excessively strong, with corresponding impulse felt on laying the hand or the ear over the præcordial region.

2. The sounds of the heart's action, and especially the first sound, less audible than natural,—obscured and muffled.

II. Of Dilatation.

1. The action of the heart weak, somewhat undulatory, with indistinct impulse.

2. The sounds of the heart's action, and especially the first, preternaturally clear, short, or abrupt.

III. Of Hypertrophy and Dilatation.

With various modifications, as the one or the other condition predominates, of the abnormal impulse, and the abnormal sounds of the heart's action.

1. The area of the natural dulness on percussion increased, often with squareness of outline of this area.

2. The apex of the heart seen and felt to beat lower down than natural, often as low as the seventh or eighth ribs.

3. The præcordial region in some cases obviously bulging and prominent.

---

*Progress and Results of Chronic Heart Disease. Prognosis.*

I. It is important to discriminate between the affection of the heart itself, and the secondary affections,—*i. e.* affections of other parts and organs resulting form it.

As time advances, there occurs a gradual aggravation of the heart affection; an increase of the valvular obstruction or of the valvular insufficiency, or of both; and likewise an increase of the hypertrophy or of the dilitation, or of both.

Sooner or later secondary affections, of various kinds, extrinsic to the heart, supervene, *e. g.*

1. Dyspnœa; bronchitis; pneumonia; pulmonary hemorrhage, with or without pulmonary apoplexy.

2. Enlargement of the liver, or of the liver and spleen; hæmatemesis.

3. Cephalalgia; tinnitus; vertigo; syncope; epilepsy; epitaxis; cerebral hemorrhage; apoplexy; palsy.

4. Dropsical effusion; œdema of face, or of face and hands,—of lungs; hydrothorax; ascites; anasarca or general dropsy.

5. Various combinations and successions of the foregoing.

II. Both the aggravation of the heart disease and the supervention of some secondary affection take place more surely and more quickly, in young and full-blooded individuals, than in those already old, or thin or emaciated.

III. As to the relation that obtains between the secondary affections and the diseased heart:—

1. The connecting link between them is the altered (abnormal)

condition of the general circulation produced by the state of the heart. This condition may be one or other, or a combination of threek inds: impeded venous current behind (pulmonic and systemic), as in mitral disease, whether obstructive or regurgitant; or undue impulsion of the arterial current in advance, as in hypertrophy; or too feeble impulsion, as in purely passive dilatation of of the ventricle.

2. Nevertheless the diseased state of the heart and these effects of it on the general circulation are commonly only *permanent predisposing* causes, not the direct or exciting causes, of those secondary affections,—the immediate causes of them (often in a great measure *avoidable*) being intemperance, over exertion and fatigue, mental emotion, and especially exposure to cold or cold and wet.

3. Some of the secondary affections are themselves the occasion, or concurrent and co-operating causes, of certain other secondary affections, *e. g.* bronchitis—of œdema of the lungs, or of general dropsy; enlargement of the liver, or of the liver and spleen—of ascites, hæmatemesis, epitaxis, apoplexy; or consolidation of lung and enlargement of the liver combined, with bronchitis—of general dropsy.

IV. The worst kind of heart affection is that in which there is at once obstructive and regurgitant disease of the mitral valve, because the most effectual in deranging the general circulation, and the most fruitful therefore of secondary affections. The least unfavorable is a diseased state of the aortic valve only, with moderate hypertrophy of the left venticle; the hypertrophy, though reckoned a diseased state, being, in fact, a provision of nature for overcoming the resistance offered to the exit of the blood.

Insufficiency of the mitral valve, however, with much hypertrophy of the left ventricle, is very hazardous; the hypertrophy in this case enhancing the evil naturally attendant on the patency of the valve.

V. When death ensues, the fatal event may take place suddenly, in the way of syncope, and be directly referrible to the diseased state of the heart; or it may take place more gradually through the medium of some secondary affection, and in very various modes,—in the way of asthenia, or in the way of coma, or in the way of asphyxia,—according to circumstances.

VI. The prognosis in any case must mainly be based upon, and may in general readily be determined from,—

1. The kind and extent of heart affection.

2. The tendency that may appear towards secondary affection; and the kind and extent of this.

3. The age and bodily habit of the patient.

4. The degree to which the habits and circumstances of the patient can be controlled and regulated, and the probable efficacy of the remedies applied, with the view in respect of them all of retarding the progress of the heart affection,—of warding off secondary offections,—and of removing those when they supervene; these several objects furnishing at the same time the main indications of treatment.—*Association Medical Journal.*

